# Low rate of non-compliance to antituberculous therapy under the banner of directly observed treatment short course (DOTS) strategy and well organized retrieval system: a call for implementation of this strategy at all DOTS centers in Saudi Arabia

**DOI:** 10.11604/pamj.2015.21.267.6280

**Published:** 2015-08-11

**Authors:** Liaqat Ali Chaudhry, Jaffar Al-Tawfiq, Ebtesam Ba-Essa, Asirvatham Alwin Robert

**Affiliations:** 1 Department of Chest Diseases, Tuberculosis Center Dammam Medical Complex (MOH) Saudi Arabia; 2 Specialty Internal Medicine, Johns Hopkins Aramco Healthcare, Dhahran, Saudi Arabia; 3 Department of Medicine & Endocrinology, Dammam Medical Complex (MOH), Saudi Arabia; 4 Department of Endocrinology and Diabetes, Diabetes Treatment Center, Prince Sultan Military Medical City, Riyadh, Saudi Arabia

**Keywords:** DOTS, antituberculous, TB-tuberculosis, non-Compliance

## Abstract

**Introduction:**

The objective of this study was to show the effectiveness of revised retrieval system on non-compliance.

**Methods:**

We retrospectively evaluated the effectiveness of a revised retrieval system on non-compliance during continuous phase of antituberculous treatment (Jan-2005 to Dec-2010) compared to baseline non-compliance (Jan-2002 to Dec-2004).

**Results:**

In the baseline period, 141 of 501 (28%) patients did not attend their first appointment. Of these 141 patients, 63 (45%) patients could be brought back to treatment while 78 patients (16%) dropped out and could not be retrieved. During the 2nd phase after launching a revised retrieval system, 98 of 835 (13%) patients did not attend their first appointment. Using the retrieval system, 79 (81%) patients were brought back for regular follow up, and 19 patients could not be retrieved, a dropout rate of 2.27%. By virtue of revised retrieval system, there was a significant drop in non-compliance by 15% and a decline in net dropout rate by 14%. The number of those brought back to treatment by revised retrieval system almost doubled (81%) compared to 44% retrieval in initial period.

**Conclusion:**

The revised retrieval system had a significant impact on the reduction of dropout rate and significant improvement in the retrieval of those patients.

## Introduction

Tuberculosis (TB) continues to be a major health problem and a leading cause of morbidity and mortality worldwide. In 2011, there were 8.7 million new cases and 1.4 million deaths from the disease, with >95% of these deaths taking place in low income and middle-income countries [1–3]. Early diagnosis and prompt treatment are essential in reducing morbidity, mortality and to break the chain of infection. The World Health Organization (WHO) in April 1993 declared tuberculosis as an emergency, and directly observed treatment short course (DOTS) strategy was launched. The main targets in 2009 were to reduce the number of new TB cases by 2015; reduce TB death rates by 50% by 2015; and at least 85% of new cases be treated successfully under directly observed treatment short course (DOTS) strategy [4, 5]. About 3 million people die of tuberculosis worldwide annually and one thousands of these are in the Kingdom of Saudi Arabia (KSA) [6]. Annual incident rate of tuberculosis has been reported recently between 14-17100,000, for Saudis 8-12.2/100,000 and 2-3 times higher in non-Saudis [7]. Tuberculosis center of Dammam medical complex is the 3rd largest governmental DOTS center in KSA. Non-compliance and increased dropout at the OPD during continuation phase of TB therapy is a preventable hurdle in the way of cure. To fight against non-compliance at our center a well-organized retrieval system backed up by local health authorities has been launched in the Eastern region in 2005. Revised retrieval system comprised of health education sessions at each step. On admission, every patient is given education on use of face mask; cough and spitting etiquette, nature of tuberculosis as a chronic disease, duration of treatment and drugs being used along their possible side effects. This was reiterated at the time of discharge, and that patients should remain adherent to treatment at home and not to miss their appointments. Patients were reminded that despite initial symptomatic relief, they need to continue uninterrupted treatment to achieve a cure. Home address and phone numbers of patients and if possible of a family member were kept as part of the record. Identifying and anticipating non-compliance in those having behavior issues and substance abuse was further managed by involvement of social worker and backing of local Government authorities.

## Methods

We retrospectively studied case files and treatment cards of all 1336 patients of open pulmonary tuberculosis admitted and treated under DOTS at the tuberculosis center of Dammam Medical Complex. Rates of non-compliance with first out-patient follow up were compared before and after implementation of a revised retrieval system. Once rendered sputum smear negative at discharge, patients were followed up in the out-patient department (OPD), during the continuation phase of therapy. The following steps were taken as part of a revised retrieval system at OPD: arranging case files and treatment cards in the OPD record room, on the basis of completed duration of treatment and according to scheduled appointments on any given date; the case files and treatment cards of those patients attending the next day are arranged one day earlier; patients who were substance abusers or had behavioral issues were reminded one day earlier of their appointments; on each visit, a special recall of their health status is given and they were reminded that for TB cure, they need to continue regular treatment and follow up; on each visit, vital signs and weight were recorded and sputum samples were taken for direct AFB smear and Mycobacterial culture as per Saudi national tuberculosis control program (SNTBCP) requirement; the results of drug sensitivity and direct sputum smear were recorded monthly; anybody not attending OPD visit was called the same day by the national tuberculosis control nurse; if a patient did not respond within two days, then social worker was involved, and for expatriates their sponsors or their company managers were involved. And if the patient was not responding after one week, then he/she was reported as a defaulter to the local authorities.

## Results

**Figure 1:**
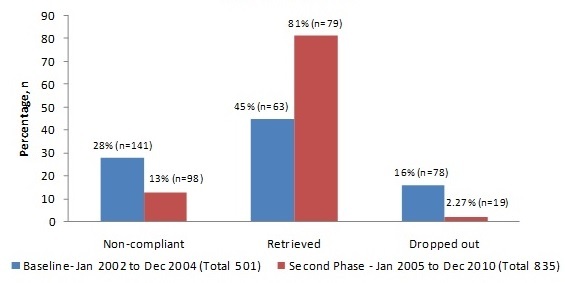
Comparison of baseline and second phase after revised retrieval system

In this study we evaluated the effectiveness of a revised well organized retrieval system with local government backing in 835 patients of pulmonary tuberculosis treated during an intensified recall system period (January 2005 and December 2010) compared to a baseline period (January-2002 to December-2004) of 501 patients (Figure 1). Table 1 shows, demographic details of all 1336 patients studied during initial phase and the 2nd phase after revised retrieval system. A total of 501 patients, treated in the initial period included males=326 (Saudis=130, Non Saudi=196); Females=175 (Saudis=40, Non Saudis=135, Housemaids=67. After revised retrieval system, a total of 835 patients were treated (males=543, Saudis=217, non-Saudis=326, Females=291 (Saudi=67, non-Saudis=223, House maids=122. Among a total of 466 female patients studied, majority 358 (77%) was non-Saudis, and out of these 189 (53%) were housemaids. During baseline phase, non-compliance and dropout rates were 28% and 16% respectively. During this phase only 45% of those non-compliant could be brought back to treatment. During 2nd phase, the rate of non-compliance and dropout rates were 13% and 2.27%, respectively. By virtue of the revised retrieval system, there was a significant reduction in the rates of non-compliance and drop-out rate by 15% and 14% respectively. The number of those retrieved and brought back to treatment during this period almost doubled (81%). The relationship of disease related aspects and non-compliance are listed in Table 2. Non-compliance was more common in re-treated cases. Reasons for being non-compliance reported in our patients are enumerated in Table 3. Of the no-compliance patients, 42 (17.57%) perceived their initial symptomatic relief as being cured. Second factor 40 (16.73%) among non-Saudi patients was administrative issues for not being permitted by their supervisors. Other significance factors were forgetfulness 26 (10.87%), long distance from the residence 23 (10.23%) and multiple co-morbidities 22 (9.20 %).

**Table 1:** Demographics of all (n=1336) patients studied

Variables in all (n = 1336) patients studied in two phases		3 years, Initial baseline Phase before revision of retrieval system Jan 2002-Dec 2004 (Total= 501 patients)	5 years, 2^nd^ Phase after revised retrieval system Jan 2005- Dec 2010 (Total= 835 patients)
**Gender**	Male	326	543
	Female	175	291
**Nationality**	Saudi	170	217
	Non-Saudi	331	135
	Housemaids	67	122

**Table 2:** Relationship of disease category and severity to non-compliance

Pattern of disease			Compliance	Non compliance	*P* value
Category	New	1309 (97.97%)	1087 (83.04%)	222 (1.95%)	<0.05*
	Re-treated	27 (2.02%)	10 (37.03%)	17 (62.9%)	
Site	Pulmonary, Extra-Pulmonary	1336 0	1097 (82.11%)	239 (17.88%)	<0.05*
Severity	*Limited	149	130	19 (7.94%=19/239)	<0.05*
	** Moderate	837	675	162 (67.78%=12/239)	
	***Advance	450	392	58 (24.26%=58/239)	
Outcome	Cured	1239 (92.73%)			
	Dropped out	97 (7.2%)			

* Limited: One lobe or less

** Moderate: >One lobe

*** Advance: Bilateral Groups compared by ‘Chi-square’ test

* *p*<0.05 consider as significant

**Table 3:** Factors for non-compliance in all (n=239) patients

No	Factors for no-compliance in our Patients	N (%)
1	Initial rapid improvement perceived as cure	42 (17.57%)
2	Long distance from residence	23 (9.62%)
3	Forgetfulness / ignorance about the disease	26 (10.87%)
4	Multiple co-morbidities	22 (9.20%)
5	Drug toxicity (continuation phase)	0
6	Paradoxical flare up of disease (one Saudi female)	1 (0.41%)
7	Superior's refusal/job related issues(low job expatriates)	40 (16.73%)
8	Non-improvement of symptoms (majority of retreated patients)	17 (7.11%)
9	Unavoidable social circumstances(exam/vacations/visiting relatives)	17 (7.11%)
10	Females dependent on their male sponsors	17 (7.11%)
11	Substance abuse/psychiatric conditions	15 (6.27%)

## Discussion

Non-compliance is defined as one or more of the following: (1) Missing > 2 consecutive weeks of DOTS, or (2) Prolongation of treatment by > 30 days due to sporadic missed doses, or (3) Poor outcome of therapy, defined as a microbiologic or clinical failure of initial therapy resulting in morbidity, relapse, or death owing to TB in defaulters (who miss two consecutive months of DOTS [8]. Tuberculosis center of Dammam medical complex is the 3rd largest government DOTS center in the kingdom of Saudi Arabia. It is a referral center for the whole of Eastern province where all patients of open TB are admitted for isolation and treated free of cost until they are rendered negative on direct smear microscopy. Availability of more affective modern antituberculous drugs being used as DOTS, has played a vital role in the treatment of tuberculosis taken for 6 months in large majority of patients. But still Non-compliance and increased dropout at the OPD during continuation phase is a preventable hurdle in the way of cure. To fight against non-compliance at our center a strong well organized retrieval system having backing from local authorities has been launched in the Eastern region from 2005 onwards. A number of risk factors for non-compliance mentioned in the present literature, both in developed as well as developing countries are, illiteracy, poverty, interrupted drug supplies, long distance from DOTS center, misperceived early symptomatic relief as cure, job related issues, drug toxicity, male gender, migration or change of residence, social stigma, alcoholism and substance abuse, and psychological factors. Substance abuse and psychological factors are common to all countries [9–11]. Various factors affecting compliance in our patients, were, misperceived initial symptomatic relief as cure, multiple co-morbidities, long distance from the center. Forgetfulness is common to all in general, Saudi patients visiting their relatives being away from their home in other city or abroad, mainly Saudis; Job related issues for not being permitted by the superiors, on vacation to home country, change of work place or transfer mainly ex-patriates; females who were not brought by their male family members mainly Saudis or sponsors for housemaids; although few in number, substance abusers and those having behavior issues were the most difficult to handle and in such cases role of local authorities was pivotal. After completing initial 2 months 4 drug intensive phase in hospital, drugs related issues were negligible during continuation phase.

During three years period between January2002 - December 2004, 141 (28%) patients missed their first appointment, 63 patients (45%) were made compliant, and 78 patients (16%) could not be retrieved with a dropout rate of 16%. After revised retrieval system, during a five years period from January 2005 - December 2010, among a total of 835 patients, 98 patients missed their appointment i.e. 13%., 79 patients (81%) were retrieved back on treatment. A total of 19 patients (2.27%) could not be retrieved. By virtue of our well organized retrieval system backed by local authorities, the number of those retrieved in January 2005 - December 2010, almost doubled (81%) as compared to only 45% during the initial phase in January 2002 - December 2004.There was significant reduction in the rate of non-compliance by 16 % and dropout rate by 14% respectively. Patients who expired, or deported expatriates having HIV and tuberculosis, expatriates who left Saudi Arabia on final exit, and those transferred to other DOTS center were excluded. In a study on non-compliance, it is observed that the treating team has been waiting for 1 to 2 weeks for the defaulters to attend on their own, before contacting them [12]. We believe 2 weeks is a very long time and is against the spirit of national tuberculosis control program (NTBCP) guidelines which advocates an uninterrupted treatment [13]. In Eastern region before implementation of DOTS strategy and lack of any retrieval system, in 1980's and late 1990's Saudi patients used to refuse hospital admissions. But now admission isolation has been made compulsory for every open case of pulmonary tuberculosis. This epidemiological move has proved to be a positive step in the right direction in breaking the chain of infection because some clusters of families has been reported having more than one patient in subsequent years. Eastern region of Saudi Arabia is one of the regions where a down ward trend of TB has been reported in the past 10 years [14]. Eastern region has a well-organized MOH medical fitness center in Dammam, where every new comer ex-patriate is screened for infectious diseases including tuberculosis before issuing residence permit (IQAMA). This center has played a great role in discovering new cases of active TB, as well as latent tuberculosis infection. Another step taken at this center is that those having latent tuberculosis are being offered treatment [15]. By adopting DOTS strategy a conversion rate of 76% at 2 months with 4 drugs intensive phase and 94% at 3 months respectively has been achieved at this center [16]. These results are in line with the goals of Saudi national tuberculosis control program. In addition to DOTS strategy during intensive phase at this center, implementation of a strong well organized retrieval system backed by local authorities has many fold enhanced compliance to continuous phase of treatment at OPD level. This has resulted in low rates of non-compliance in general and has grossly reduced the rates of dropout in particular. Well organized retrieval system if backed by the local authorities become a very effective tool ensuring a successful national tuberculosis control program and should be adopted at national level as an integral part of NTBCP.

## Conclusion

The revised retrieval system had a significant impact on the reduction of dropout rate and significant improvement in the retrieval of those patients.
